# Spectral analysis comparison of pushbroom and snapshot hyperspectral cameras for *in vivo* brain tissues and chromophore identification

**DOI:** 10.1117/1.JBO.29.9.093510

**Published:** 2024-09-24

**Authors:** Alberto Martín-Pérez, Alejandro Martinez de Ternero, Alfonso Lagares, Eduardo Juarez, César Sanz

**Affiliations:** a Universidad Politécnica de Madrid, Research Center on Software Technologies and Multimedia Systems, Madrid, Spain; b Hospital Universitario 12 de Octubre, Neurosurgery Department, Madrid, Spain; c Universidad Complutense de Madrid, Surgery Department, Medicine Faculty, Madrid, Spain; d Instituto de Investigación Sanitaria Hospital 12 de Octubre (imas12), Madrid, Spain

**Keywords:** hyperspectral imaging, hyperspectral snapshot camera, *in vivo* brain tumor, spectral camera comparison, human brain, brain cancer

## Abstract

**Significance:**

Hyperspectral imaging sensors have rapidly advanced, aiding in tumor diagnostics for *in vivo* brain tumors. Linescan cameras effectively distinguish between pathological and healthy tissue, whereas snapshot cameras offer a potential alternative to reduce acquisition time.

**Aim:**

Our research compares linescan and snapshot hyperspectral cameras for *in vivo* brain tissues and chromophore identification.

**Approach:**

We compared a linescan pushbroom camera and a snapshot camera using images from 10 patients with various pathologies. Objective comparisons were made using unnormalized and normalized data for healthy and pathological tissues. We utilized the interquartile range (IQR) for the spectral angle mapping (SAM), the goodness-of-fit coefficient (GFC), and the root mean square error (RMSE) within the 659.95 to 951.42 nm range. In addition, we assessed the ability of both cameras to capture tissue chromophores by analyzing absorbance from reflectance information.

**Results:**

The SAM metric indicates reduced dispersion and high similarity between cameras for pathological samples, with a 9.68% IQR for normalized data compared with 2.38% for unnormalized data. This pattern is consistent across GFC and RMSE metrics, regardless of tissue type. Moreover, both cameras could identify absorption peaks of certain chromophores. For instance, using the absorbance measurements of the linescan camera, we obtained SAM values below 0.235 for four peaks, regardless of the tissue and type of data under inspection. These peaks are one for cytochrome b in its oxidized form at λ=422  nm, two for ${\mathrm{HbO}}_{2}$ at λ=542  nm and λ=576  nm, and one for water at λ=976  nm.

**Conclusion:**

The spectral signatures of the cameras show more similarity with unnormalized data, likely due to snapshot sensor noise, resulting in noisier signatures post-normalization. Comparisons in this study suggest that snapshot cameras might be viable alternatives to linescan cameras for real-time brain tissue identification.

## 1 Introduction

Cancer remains one of the leading causes of morbidity and mortality in the world, with ∼18.1 million new cases of cancer diagnosed worldwide in 2020. In addition, the International Agency for Research on Cancer estimates that by 2040 the diagnosis of new cases will increase to 27.0 million.^1^ In Spain, 1.49% of newly diagnosed cancers in 2022 (4.169 of 280.101) were tumors of the encephalo or nervous system.^1^ Identifying pathological tissue from healthy tissue is challenging, especially with aggressive tumors such as grade IV glioblastoma (GB) that have high infiltration capabilities.^2^ In addition, GB has poor long-term survival rates,^3^ making surgery an unavoidable process to increase patient survival. However, the brain shifting toward the skull opening can result in cerebrospinal fluid leakage and hinder tumor identification due to the alterations in the structure of the surrounding tissue, rendering preoperative imaging inadequate for intra-operative conditions.^4^ Therefore, intraoperative tools for brain tumor surgery are essential to help neurosurgeons in delineating and locating the tumor. Neuronavigators are precise instruments that facilitate the real-time monitoring of surgical interventions through the use of magnetic resonance imaging (MRI) or computed tomography scans conducted prior to the procedure. Nonetheless, they present limitations in pinpointing its exact location once the brain is exposed.^5^ Solutions that have been developed to address the issues associated with neuronavigators include the use of intraoperative MRI. This has the advantage of being able to locate the tumor after the craniotomy, thereby solving the problem of brain shift. Nevertheless, it should be noted that the use of MRI increases the time required for surgery and that it requires the use of specific equipment during the surgical procedure.^6^^,^^7^ Another tool that is not affected by the brain shift issue that operates in real time at a low cost is the intraoperative ultrasound. However, the data must be interpreted by experienced users since the images are of low resolution.^8^^,^^9^ Fluorescent tumor markers from add-on agents such as 5-aminolevunilic acid are able to deliver highly accurate intraoperative tumor margin detection with a rapid refresh rate. Yet, these are invasive methods since they require the injection of the agent^10^ into the patient and present a limited ability to define the tumor margin during surgery for low-grade gliomas.^11^

Therefore, faster and non-invasive techniques, compared with the tools described previously, are crucial to the success of surgical interventions. A widely used non-invasive and non-ionizing technique that requires no contact with patients is hyperspectral (HS) imaging (HSI).^12^ The advancement in HS sensors in the past years and the variety of available options can make the decision difficult of which HS camera to use. In particular, HS cameras are capable of capturing both spatial and spectral data using various techniques,^13^ scanning-based (SB) and wide-field (WF) being the typical imaging methods for HSI. First, SB approaches can acquire the spectrum for each pixel using whiskbroom (point-scanning) instruments, a line of pixels in pushbroom (line-scanning) instruments, or using a wedge filter that disperses light spectrally along one dimension (wedge-scanning). Second, WF approaches capture the whole scene in a single exposure with 2D detector arrays, either by stepping through the wavelength spectrum to complete the data cube (wavelength scan) or by acquiring the spatial and spectral information at the same time (snapshot). However, recent techniques such as snapscan cameras,^14^ which combine SB and WF approaches, provide compact solutions with faster acquisition times than linescan cameras while offering higher spatial and spectral resolution than snapshot sensors. It is worth noting that the most used spectral range in medical applications falls in the visible (VIS) (400 to 780 nm) and near-infrared (NIR) spectrums (780 to 2500 nm).^12^ Regardless of the possibilities available to select HSI equipment, pushbroom linescan, snapscan, and snapshot HS cameras have been used as part of intraoperative tools in several studies to differentiate brain tumors from healthy tissue in *in vivo* human brains.^15^^–^^18^ For example, Fabelo et al. developed an intraoperative acquisition system based on two pusbroom linescan cameras, a visible and near-infrared (VNIR) and a NIR in the spectral range between 400 to 1000 nm and 900 to 1700 nm, respectively.^15^ The intraoperative acquisition and data processing took ∼1  min, which does not enable real-time solutions understood as providing a live sequence of images. Furthermore, other works by Vandebriel et al. have assembled a snapscan HS camera to a surgical microscope for improving the removal of low-grade gliomas.^16^ The camera captured 104 spectral bands between 470 and 787 nm since it had to match the intrinsic illumination of the microscope. Even though the snapscan camera can provide high spatial resolution in a wide spectral range and reduce the acquisition time to less than 3 s for static targets,^14^ it requires an internal movement of a linescan sensor to acquire an HS cube, which is not suitable for real-time solutions. Moreover, recent works have developed an intraoperative system based on HSI with a pushbroom linescan and a snapshot HS cameras^18^^,^^19^ for brain tumor detection. On one hand, the snapshot camera captures 25 spectral bands in the 660 to 950 nm spectral range with a spatial dimension of 409×217  pixels each band. On the other hand, the pushbroom linescan camera acquires 369 bands between 400 and 1000 nm with a spatial dimension of 1600 pixels on each line. Despite the low number of bands acquired by the snapshot camera and its low spatial resolution, it can enable real-time solutions such as live video classifications with machine learning (ML) algorithms.^19^

Therefore, this research study aims to compare two different HS cameras that differ in how they acquire data, a pushbroom linescan and a snapshot, to determine their potential use for brain tissues and chromophore identification. To compare how similar their signatures are, the analysis is conducted in the NIR spectrum, specifically in the 659.95 to 951.42 nm range. The study involved examining and using ten different *in vivo* human brain images from the Slim Brain database^20^ to provide useful results for biomedical purposes. All the brains of patients used from the database were captured with both HS cameras to ensure similar lighting conditions for the comparisons.

## 2 Materials and Methods

### 2.1 Hyperspectral Camera Specifications

Two different HS cameras based on different acquisition techniques have been used to capture *in vivo* brain images. The technical specifications of both cameras, sensors, and lenses are presented in Table 1.

**Table 1:** Sensor and camera optics specifications of the different HS cameras used. The last five parameters are fixed during the acquisition of images in the operating room.

Camera	Snapshot 5×5-mosaic	Pushbroom linescan
Sensor	CMOS	sCMOS
Sensor ADC (bits)	8	16
Pixel size (μm)	5.5	6.5
Sensor resolution (px)	2048×1088	1600×1
Output resolution (px)	2045×1085	1600×1
Band resolution (px)	409×217	1600×1
Effective no. of bands	25	369
Spectral range (nm)	660 to 950	400 to 1000
Bandwidth (FWHM avg.) (nm)	15	5.8
Focal length (mm)	35	35
F-number	4	2
Frame period (ms)	110	160
Exposure time (ms)	100	150
Working distance (cm)	60	60

On one hand, the snapshot HS camera has a complementary metal oxide semiconductor (CMOS) sensor holding a 5×5-mosaic pattern with a pixel size of 5.5  μm (MQ022HG-IM-SM5X5-NIR, Ximea GmbH, Münster, Germany). The analog to digital converter (ADC) of the sensor provides images with a resolution of 8 bits. In addition, a long pass filter (FELH0650, Thorlabs, Inc., Newton, New Jersey, United States) with a 650-nm cut-on wavelength is placed in front of the lens to remove non-negligible secondary harmonics, which were determined by the manufacturer in the spectral response curves during sensor production. Although the sensor resolution is 2048×1088  pixels, the active area of the sensor with the built-in spectral filters has 2045×1085  pixels. This reduced area is called the active filter zone, which omits the last three rows and last three columns from the total sensor resolution. Each capture with this snapshot camera produces an image containing the spatial and spectral resolution due to the 5×5-mosaic pattern. Each mosaic contains approximately the same spatial pixel at different 25 wavelength bands within the NIR spectrum, specifically in the 660 to 950 nm spectral range. In addition, these bands are spaced among each other with a mean and standard deviation value of 12.11±2.64  nm. Hence, the mosaic pattern reduces the 2D output spatial resolution by a factor of 5 to obtain a 3D HS cube. In particular, the image with 2045×1085  pixels generated by the sensor is arranged into a 409×217×25 HS cube to perform the spectral analysis. The main advantage of the snapshot camera is its capability for real-time solutions, understood as processing a sequence of HS images to provide a live video of the scene. On the other hand, the other camera is based on the pushbroom linescan technology (Micro-Hyperspec^®^ E-Series, HeadWall Photonics Inc., Bolton, Massachusetts, United States), which holds a scientific CMOS sensor with an ADC of 16 bits and a pixel size of 6.5  μm. The sensor acquires a single-spatial line with 1600 pixels and 394 wavelengths of information. Thus, the camera needs to be moved with an actuator to scan an image with as many lines as desired. All *in vivo* brain captures used in this study were scanned with 500 lines, producing images with a spatial resolution of 1600×500  pixels and 394 spectral bands. Besides, the exposure time and frame period were set to 150 and 160 ms, respectively. Although the sensor is sensitive in the 365 to 1004 nm spectral range, those wavelengths acquired outside the 400 to 1000 nm spectral range need to be removed, as specified by the manufacturer. Eliminating such bands results in 369 effective wavelength bands separated by 1.62±0.00  nm from each other.

It is worth noting that captures from both cameras were cropped spatially to help neurosurgeons during the labeling processing. Therefore, the spatial resolution of the HS linescan or snapshot captures are smaller than 1600×500  pixels or 409×217  pixels, respectively. Although the pusbroom linescan camera provides more spatial resolution and spectral information, it is not suitable for real-time solutions due to the scanning procedure. The time spent to scan a brain image requires, ∼1  min and 40 s, whereas an image captured with the HS snapshot camera takes 100 ms.

### 2.2 Acquisition System

**Fig. 1:**
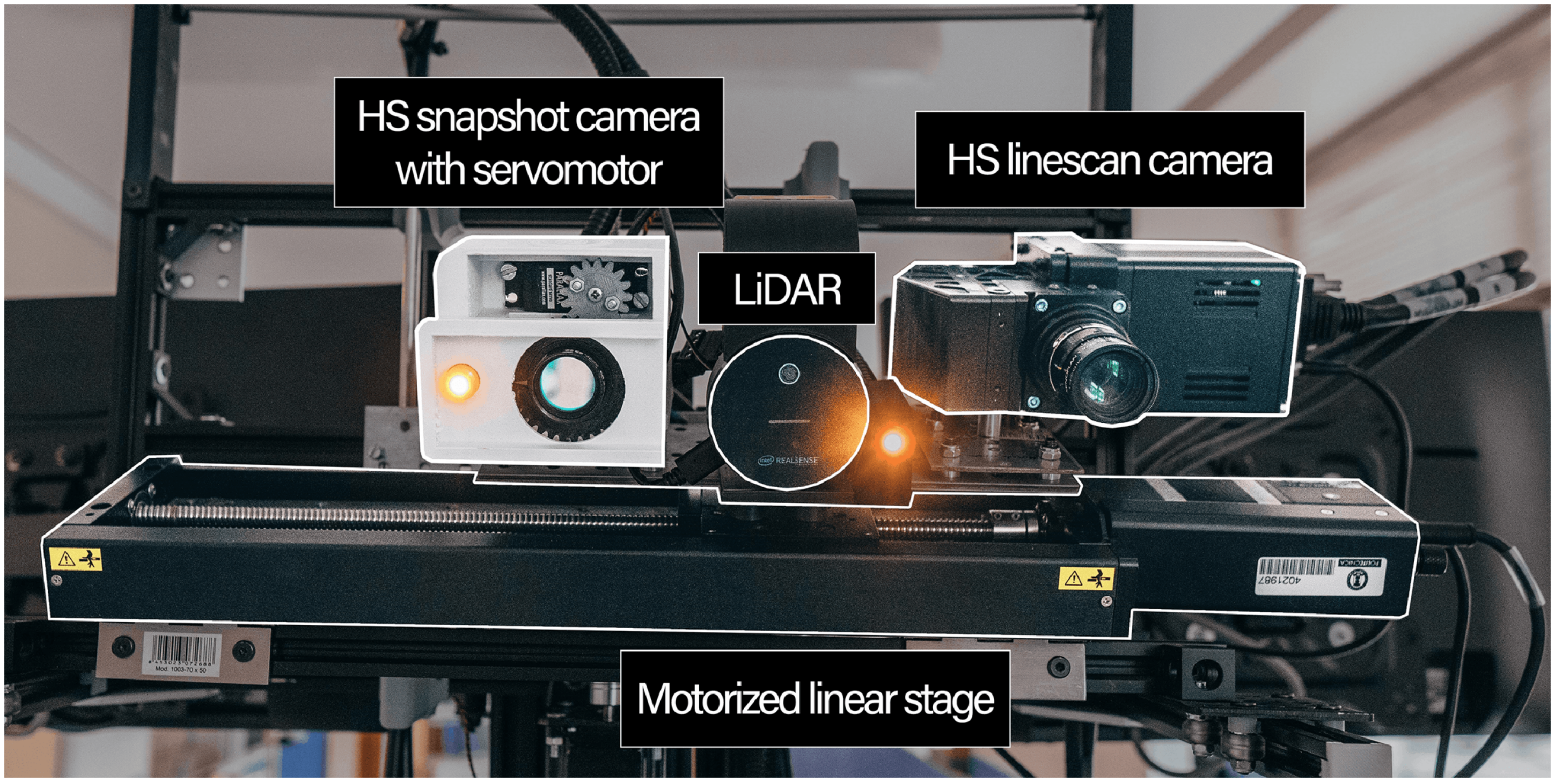
Front view of real acquisition system with main components highlighted and labeled.

The acquisition system used to gather the *in vivo* brain images is presented in Fig. 1. Starting from the left, the HS 5×5-mosaic snapshot camera is located inside a 3D-printed white case. Inside the case, there is a servomotor employed to help focus the camera. An external light source with a 150 W 21 V EKE halogen lamp (MI150, Dolan-Jenner, Boxborough, Massachusetts, United States) and a dual gooseneck fiber optic (EEG28, Dolan-Jenner) were used to illuminate the brains. While the housing of the light source does not appear in the image, the dual fiber optics are shown glowing in Fig. 1. The system has been utilized as a real-time augmented reality (AR) application through laser imaging detection and ranging (LiDAR) equipped with an RGB camera. This technology enables the collection of geometric data of the brain surface by extracting a point cloud from the scene using depth and RGB information. The manufacturer indicates that the depth accuracy of the system depends on reflectivity and light conditions and is effective within a range of 0.5 to 3.86 m. Specifically, the random error, measured as the standard deviation, is ≤17  mm, whereas the typical systematic error is <11  mm plus 0.1% of the measured distance, provided there is no multi-path interference. By combining depth information from the point cloud, ML classification results on HS data, and the RGB from the LiDAR camera in an AR interface, the effectiveness of surgical field exploration and tumor delineation can be enhanced.^19^ In this study, LiDAR is specifically used to measure the distance between the acquisition system and the brain surface. This measurement helps to focus the system accurately by determining the distance to a single point on the brain surface. Given that the linescan camera has a fixed focusing distance of 60 cm, the measurement of the LiDAR is necessary to ensure focused captures with the linescan camera. Therefore, all images were taken at a distance of 60 cm to ensure a fair comparison between the two cameras, taking into account the limited focus distance of the linescan camera. Furthermore, the motorized linear stage (X-LRQ300HL-DE51, Zaber Technologies Inc., Vancouver, Canada) below the imaging sensors is mainly used to move the HS linescan camera. This movement allows the scanning of the brain image to compose a HS cube with the desired spatial resolution. The sensors and fiber optics are all aligned in the same plane, which is perpendicular to that of the linear stage holding them. Although not included in Fig. 1, two additional motorized linear stages are used to tilt and provide height to the stage.

### 2.3 Data Processing

All HS images, regardless of the camera used, are pre-processed using almost the same procedures, which are presented in Table 2.

**Table 2:** Pre-processing steps performed on the images captured with the different HS cameras used.

Camera	Snapshot 5×5-mosaic	Pushbroom linescan
1. Radiation calibration	✓	✓
2. Cube formation	✓ (Rearrange of 2D image)	✓ (Line stitching)
3. Spectral correction	✓	✗
4. Bands removal	✗	✓ (Extract effective bands)
5. Noise filtering^21^	✓	✓
6. Normalization	✓	✓

The first step is to obtain the reflectance information of the material interrogated by calibrating the raw information obtained with the sensor. Equation (1) eliminates the effect of the HS sensor and the lighting conditions captured with the raw images to obtain the reflectance information R from the sample,

$$R=\frac{I_{\text{raw}}-I_{\text{dark}}}{I_{\text{white}}-I_{\text{dark}}},$$ (1)

where $I_{\text{raw}}$ is the captured raw data of the sample, $I_{\text{dark}}$ is the raw dark reference captured with the lens cap in front of the camera lens, and $I_{\text{white}}$ is the raw white reference intensity reflected over a Lambertian diffuse target with 95% of reflectance values (SG 3151-U, SphereOptics GmbH, Herrsching am Ammersee, Germany). All white references are captured under the same conditions as the images captured, meaning that each calibrated image needs a $I_{\text{white}}$ captured with the same working distance and tilt angle. While $I_{\text{dark}}$ is used to remove the ambient temperature and electrical noise introduced to the measurement, $I_{\text{white}}$ tries to reduce the influence of the light sources on the sample. It should be noted that prior to imaging the brains, the exposure times of both cameras were evaluated to ensure that no measurements on $I_{\text{white}}$ were saturated.

The second step is related to the formation of the HS cubes. The snapshot sensor captures 2D images that need to be rearranged into a 3D cube, which has a spatial dimension five times smaller than the 2D images due to the mosaic pattern of the sensor described in Sec. 2.1. However, the pushbroom camera has a simpler process to conform an HS cube. Such a process is called line stitching, which requires a precise linear actuator to move the HS camera to join adjacent spatial lines while avoiding overlap. The spectral correction is the third step and only applies to the snapshot camera. This correction is necessary to correct the response curves of the HS snapshot sensor, which presents crosstalks between adjacent pixels of the sensor that vary with the angle of incident light to the sensor.^22^ To correct this effect, the manufacturer provides a spectral correction matrix that modifies the response of the sensor to obtain ideal Gaussian curves. The spectral correction process is described in Eq. (2)

$$R_{\mathrm{sc}}=R\times \mathrm{SCM},$$ (2)

where $R_{\mathrm{sc}}$ is the spectrally corrected reflectance data, R is the reflectance of the sample after using Eq. (1), and SCM is the spectral correction matrix. The fourth step is the removal of bands, which only applies to the pushbroom camera. As described in Sec. 2.1, the sensor captures more information than what can be effectively used. Briefly, this process eliminates the spectral bands not taken between the 400 and 1000 nm spectral range. The fifth step consists of applying the noise filtering algorithm of HySime, which was presented by Bioucas-Dias et al.^21^ We specifically use the noise estimation procedure of HySime and assume that the noise in the HS cube is additive. In such a procedure, HySime deduces the noise present in an HS cube by making the assumption that the reflectance at a specific band can be effectively characterized through linear regression using the remaining bands. After estimating the noise in an HS cube, we employ this estimation to subtract the noise from the original HS cube, thus carrying out the noise-filtering process for each image independently. The last step is a normalization process that homogenizes the spectral signatures to help compare captures. For this study, the normalized reflectance $R_{\text{norm}}$ is obtained using a min-max normalization for every spectral pixel independently, as described in Eq. (3), which forces data to a range of 0 to 1

$$R_{\text{norm}}=\frac{R_{\mathrm{sc}}-\min(R_{\mathrm{sc}})}{\max(R_{\mathrm{sc}})-\min(R_{\mathrm{sc}})},$$ (3)

where $R_{\mathrm{sc}}$ is the spectrally corrected reflectance data obtained with Eq. (2) for the HS snapshot camera or the reflectance information R obtained with Eq. (1) for the HS linescan camera. For further clarification, in Eq. (3), the noise filtering is applied to either $R_{\mathrm{sc}}$ or R before applying the normalization.

### 2.4 In Vivo Human Brain Images

**Fig. 2:**
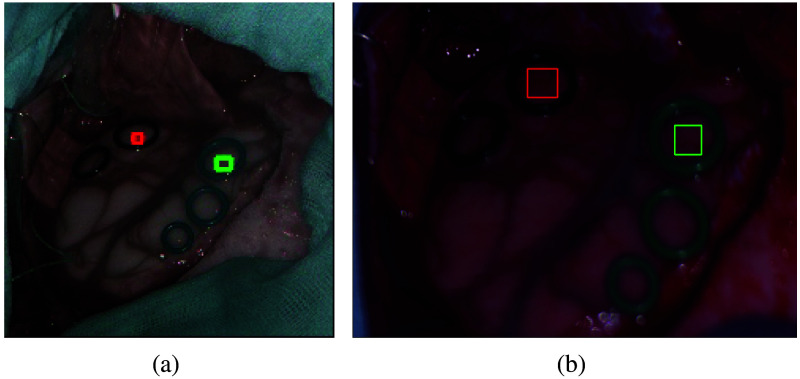
Pseudo-RGB with healthy and pathological ROIs for patient ID 190. (a) Patient ID190-Snapshot. (b) Patient ID190-Linescan.

For this study, we used 10 *in vivo* brains from adult patients who have provided informed consent prior to surgery. These 10 patients were selected because two or more sterilized rubber rings were placed by neurosurgeons prior to acquire the HS images. Different colors were used for tissue identification, with green and black being used for healthy and pathological tissues, respectively. These rubber rings can be seen in the pseudo RGBs of Fig. 2 for patients with ID 190. In addition, the same images for the rest of the patients are presented in Fig. S1 in the Supplementary Material. As indicated previously, images of patients who have suffered different pathologies have been used. More specifically, patients with ID 177, 183, 184, 190, 193, and 203 have GB with isocitrate dehydrogenase (IDH) non-mutated. Patient ID 185 has grade II astrocytoma with IDH mutation, patient ID 192 has a metastatic testicular tumor, and patient ID 194 has a metastatic lung carcinoma tumor. Finally, patient ID 201 exhibits pseudoprogression in a GB with IDH non-mutated, indicating apparent GB progression likely attributable to treatment effects rather than actual tumor growth.

The guidelines of the Declaration of Helsinki have been followed, and the acquisition of HS images has been approved by the Research Ethics Committee of Hospital Universitario 12 de Octubre, Madrid, Spain (protocol code 19/158, May 28, 2019). All patients shaved the area to be operated on prior to the scalp incision. Then, a high-speed drill was employed to make burr holes in the skull, which are used to insert a cranial drill to perform the craniotomy. This procedure extracts a bone flap to expose the dura of the patient, and then, the durotomy is performed by cutting with a knife the dura to uncover the brain surface. Then, both HS cameras proceeded to acquire the *in vivo* brain surface.

Using the rubber rings ensures that the data of both cameras comes from the same spatial location of the brain surface. Furthermore, this procedure is similar to the one employed by Mühle et al.,^23^ who used a plastic cursor to delimit a bordered region of interest (ROI) over biological organs to compare different HS cameras. In addition, the biomedical experts selected similar areas of size 21×21  pixels inside the plastic cursor to perform their analysis. In our case, the areas we selected inside the rubber rings have a size of 5×5  pixels since bigger ROIs got pixels outside the rings in the snapshot images. Although in the previous study, Mühle et al. took 10 HS snapshot images to average them; in our study, it was not feasible since the *in vivo* human brain is in motion due to the heartbeat. Moreover, the linescan camera is inevitably affected by the motion of the brain during the scanning procedure, hindering the possibility of averaging multiple captures. For these reasons, one HS snapshot and one HS linescan image were taken for each patient. A single linescan image takes ∼1  min and 40 s, whereas a snapshot measurement takes 100 ms. Notice that specular reflections have not been removed, as neither the cameras nor the light source had polarizers. Although the patients exhibit diverse pathologies, the spectral comparisons are conducted on an intra-patient basis. This entails the comparison of the spectral signatures of both cameras for each patient individually.

### 2.5 Spectral Similarity Metrics

A way to compare both HS cameras is to employ spectral similarity metrics, which can assess how different reflectances are related to each other. Agarla et al. have examined 14 frequently used measures and grouped them into five categories based on the type of error they evaluate,^24^ including the mathematical definition and implementation of all metrics. Furthermore, as stated by Agarla et al., selecting one measure for each of the groups, they describe can be sufficient to assess the spectral similarity.^24^ For that reason, we decided to use the root mean square error (RMSE), the goodness-of-fit coefficient (GFC),^25^ and the spectral angle mapper (SAM)^26^ metrics. On one hand, RMSE can range from 0 to 1 since the maximum value of our data is 1, indicating a value of RMSE equal to 0 perfect similarity. On the other hand, GFC values can be between the 0 and 1 range, where a value of 1 indicates complete similarity. SAM values range from 0 to 1, indicating values closer to 0 with high similarity and values closer to 1 with low similarity.

**Fig. 3:**
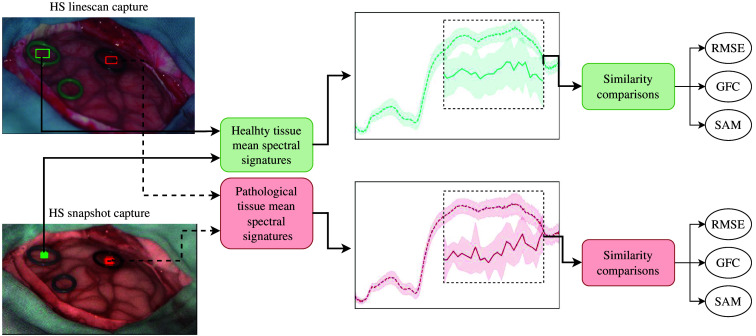
Procedure to compute the spectral similarities acquired with both HS cameras. The comparison is made independently by patient and tissue, using the spectra shared by both cameras.

These metrics are chosen since they are applicable to the spectral domain, can be used as loss functions (except SAM since angular metrics is not used to measure losses), and do not need extra requirements to be computed. Furthermore, the spectral signatures of the HS linescan camera are considered as the reference spectra, whereas those obtained with the HS snapshot camera are considered as the arbitrary signal. The previous assumption is grounded on the fact that the HS linescan camera gathers greater spatial and spectral data compared with the snapshot camera. We compute and compare the mean spectral signatures as illustrated in Fig. 3. For each patient, we independently obtain the mean spectral signatures of the pixels in the ROIs located inside the rubber rings, as shown on the left side of Fig. 3.

Since the HS snapshot camera captures less spatial resolution, less pixels are included in the green and red ROIs inside the rubber rings compared with those captured by the HS linescan. Specifically, the ROIs created for all snapshot captures have a 5×5 spatial dimension. Therefore, to provide a fair comparison, we created bigger ROIs for the HS linescan but randomly selected 25 pixels. Then, with those 25 pixels for each tissue and camera, the mean spectral signature and standard deviation are computed as presented with the plots in the middle of Fig. 3, which include black dashed rectangles to indicate the part of the spectrum shared by both cameras and used to compare them with the spectral similarity metrics. Finally, the RMSE, GFC, and SAM metrics are computed for each tissue using the matched wavelengths of the cameras presented in Table S1 in the Supplementary Material.

### 2.6 Analyzing Absorbance Measurements to Identify Chromophore Absorption Peaks

To analyze the absorbance (A) spectral signatures and look for patterns that might indicate the presence of certain chromophores, we will use the reflectance (R) measured by the cameras. A is commonly derived,^27^^–^^29^ for each wavelength, from R using Eq. (4)

$$A(\lambda )=-\log_{10}(R(\lambda )).$$ (4)

This expression is derived from the Beer-Lambert law,^30^ which expresses A as $A=\log_{10}(I_{o}/I)$. Here, $I_{o}$ represents the incident light, and I is the light that has passed through the sample. In our specific context, we are dealing with reflectance information as defined in Eq. (1). The maximum reflected light corresponds to $I_{\text{white}}$ (analogous to what would be $I_{o}$ in the Beer-Lambert law), and $I_{\text{raw}}$ represents the light that has passed through the brain (similar to I in the Beer-Lambert law). To account for the noise of the sensor, we include the dark measure from Eq. (1) ($I_{\text{dark}}$). This allows us to mitigate sensor noise, and as a result, we arrive at Eq (4) $A=\log_{10}((I_{\text{white}}-I_{\text{dark}})/(I_{\mathrm{raw}}-I_{\text{dark}}))=\log_{10}(1/R)=-\log_{10}(R)$.

The chromophores we will attempt to identify are those that contribute the most to absorb light in the NIR region (from 800 to 2500 nm) in adult brains. As stated by Correia et al., these are hemoglobin, water, lipid, and the following cytochromes: cytochrome aa3 (Cyt aa3), cytochrome b (Cyt b), and cytochrome c (Cyt c).^31^ The absorption spectra of the previous cytochromes on their redox state between 400 and 1000 nm were obtained from the Biomedical Optics Research Laboratory (BORL) Github repository.^32^ It is worth noting that we converted the molar extinction coefficient for the Hb to the absorption coefficient as specified by Prahl et al.,^33^ considering that for the whole blood, there is 150 g of Hb per liter. Although this assumption may be doubtful for the measurements taken, it allows us to visually compare the spectrum of all chromophores with the same units. Also, it is worth noting that the absorbance, A, and the absorption coefficient, $\mu_{a}$, represent different phenomena. On one hand, A is a property of a material that measures the fraction of light that can pass through in terms of intensity. On the other hand, $\mu_{a}$ is a property of the material that describes its effectiveness in absorbing light. We know from the Beer-Lambert law^30^ that A and $\mu_{a}$ are related through Eq. (5):

$$A(\lambda )=\epsilon \times c\times l=\mu_{a}\times l,$$ (5)

where ϵ is the molar absorptivity, also called the extinction coefficient, with units of $\frac{L}{\mathrm{mol}}\;{\mathrm{cm}}^{-1}$, c is the concentration of a solution in the sample in $\frac{\mathrm{mol}}{L}$, l is the length of the sample that light passes through in cm, and $\mu_{a}=\epsilon \times c$ is the absorption coefficient in ${\mathrm{cm}}^{-1}$. Unfortunately, l is unknown for the brain images used in this study. Therefore, we cannot convert the A measured with the HS cameras to $\mu_{a}$, for comparing the chromophores spectra with the absorbance measurements of the cameras. However, we can use the SAM metric described in Sec. 2.5 to try to identify the $\mu_{a}$ peaks of the chromophores in the A measurements. Of all the metrics used in this study, SAM is the only one that focuses on the shape of the spectra rather than the numerical values.^24^ Although we cannot indicate the percentage concentration of each chromophore in the camera measurements, we try to identify their peaks by selecting wavelengths that include the $\mu_{a}$ peak wavelength and those around it. The most relevant absorption peaks of each chromophore are presented in Fig. S2 in the Supplementary Material.

## 3 Results

### 3.1 Diffuse Reflectance Standard Measurements

**Fig. 4:**
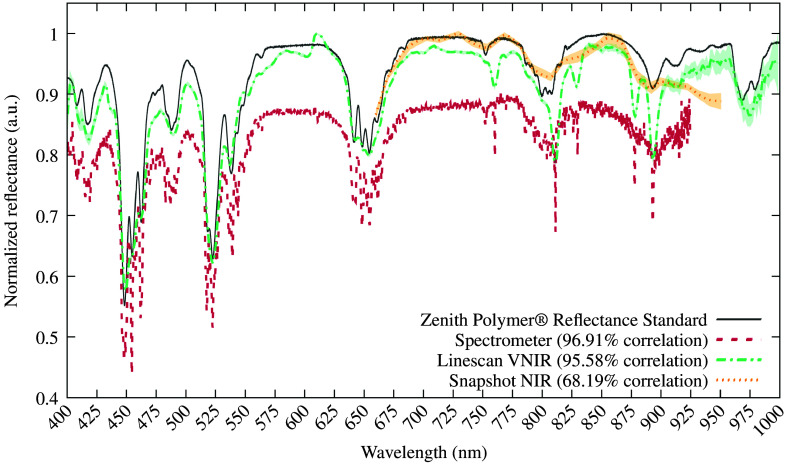
Normalized reflectance spectral responses when the zenith polymer reflectance standard was illuminated with different sensors. The spectrometer measurement was conducted with a fiber optic positioned orthogonally to the polymer reference at a distance of 5 cm. The HS camera measurements were obtained by capturing a scene of the polymer at ∼40.5  cm distance, utilizing an ROI of 25×25  pixels. The responses from the HS cameras represent the mean spectral signatures of the polymer pixels in the ROI, with their corresponding standard deviation, as indicated by the shaded color between the mean and the corresponding data points. The spectrometer employs 1913 bands and encompasses the 400 to 925 nm spectrum. The HS linescan VNIR camera covers the 400 to 1000 nm spectral range using 369 bands. Finally, the HS snapshot NIR camera spectrum range covers from 660 to 950 nm using 25 bands. The Pearson correlation coefficient is presented after comparing the measured bands of each sensor with the polymer reference response.

To validate the spectral performance characterization of the HS cameras and enhance the reliability of the subsequent analysis of the patient data, reference measurements were conducted on the Zenith Polymer Reflectance Standard, which exhibits nearly ideal Lambertian 99% of diffuse reflectance (SphereOptics GmbH, Herrsching am Ammersee, BY, Germany). The spectral response of the polymer reference is provided by the manufacturer and is illustrated with a gray line in Fig. 4. Moreover, the Pearson correlation has been used between the system measurements and the reference polymer signature to evaluate the performance of the HS cameras. Furthermore, a miniature spectrometer (Ocean Insight, Orlando, Florida) was employed to analyze the spectral responses of the polymer reference. This device is capable of measuring in the visible (VIS) and near-infrared (NIR) spectrum from 350 to 925 nm, with an ADC resolution of 16 bits. For the purposes of this study, measurements taken with the spectrometer from 400 to 925 nm are presented with a red dashed line in Fig. 4, as this includes the spectral range under analysis in the following sections. The correlation obtained when using 1913 bands from the spectrometer is 96.91%. In the same figure, the results of the measurements taken with both HS cameras are illustrated with green dashed-dotted lines and orange dotted lines for the linescan and snapshot cameras, respectively. The correlation obtained with the linescan camera using 369 bands is 95.58%, whereas for the snapshot camera, it is 68.19% using 25 bands. Although the correlation with the snapshot camera is lower than that obtained with the spectrometer or the other HS camera, the orange line clearly demonstrates that the spectral response is relatively similar between 660 and 866 nm, with a Pearson correlation value of 95.55% using the 17 bands in the aforementioned range.

### 3.2 Brain Tissue Measurements

**Fig. 5:**
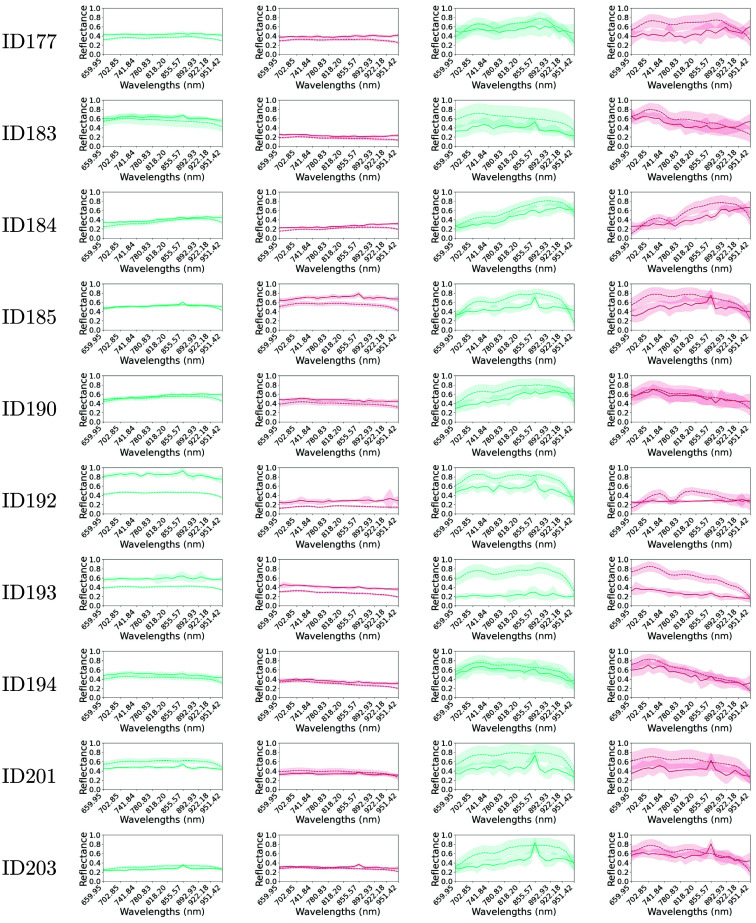
Mean spectral signatures with standard deviation for every patient in the study. Data comes from the 25 pixels included inside the rubber rings captured with both HS cameras. Continuous curves are from the snapshot camera, whereas dashed line curves are from the linescan camera. Plots are in green and red to represent the healthy and pathological tissues, respectively. The two columns on the left are the spectral signatures when data has been calibrated and denoised, whereas in the two columns to the right, data has additionally been normalized using a min-max normalization.

The reflectance measurements obtained with both HS cameras from the 10 *in vivo* human brains are shown in the Supplementary Material, specifically in Fig. 5. The illustrated spectral signatures have been obtained, for every camera measurement, using 25 pixels located inside the rubber rings as shown in the images in Fig. 2. We decided to analyze the effect of normalizing the calibrated and denoised data to check how it would influence on the spectral similarity metrics. Hence, the two columns to the left in Fig. 5 are data that have been calibrated and denoised, whereas the two columns to the right are the same data that have been normalized using Eq. (3). The spectral range analyzed to address the comparison is between 659.95 and 951.42 nm using 25 bands detailed in Table S1 in the Supplementary Material for each camera. When looking at the unnormalized data, similar spectral signatures are measured with both HS cameras for eight out of the 10 *in vivo* human brains. Note how the spectral signatures of both cameras for patients 192 and 193 are less similar compared with other patients for either the healthy or pathological tissues. Furthermore, the healthy tissue measurements from the different patients have values between 0.2 and 0.6 in most cases, excluding patient 192, which has values between 0.4 and almost 1.0. Moreover, the reflectance values for pathological tissues range from 0.1 to 0.4 for most patients, excluding patient 185 whose values are between 0.5 and 0.8. The variations in reflectance or spectral signatures could result from the biological differences between the patients and their distinct brain tumor pathologies. Overall, the mean spectral signatures of both cameras differ more from each other once the normalization is applied. For instance, the healthy tissue measurements of patient 183 show a 0.3 reflectance difference between the spectral signatures of both cameras, whereas the unnormalized data for the same patient has a variation of less than 0.05. Such behavior can be seen for most patients and tissues except the healthy spectral signatures of patient 192 because it already exhibits a great difference in the unnormalized measurements.

In addition, we can see an increase in the standard deviation shown in shaded colors around the mean curves due to the normalization. It is worth noting that the location of each pixel on the curved brain surface implies light variations, leading to slightly different measured reflectances. This, in turn, increases the difference between pixels within the same ROI when normalizing the reflectance to a range between 0 and 1. While measurements between cameras display a consistent trend across patients and tissues, snapshot measurements show more noise after normalization compared with the smoother linescan camera measurements. This results in more pronounced peaks and valleys in the spectrum of the snapshot measurements. However, there is a common peak across most measurements at λ≈850  nm, which is influenced by the laser used by the LiDAR while capturing only with the snapshot camera. Such a peak can be seen in patient 203 measurements in Fig. 5. Variations of the amplitude of such peak are due to the different angles at which the patients were captured. By comparing measurements with and without normalization, it is worth noting that the aforementioned peak at λ≈850  nm is more pronounced once the normalization is applied.

**Fig. 6:**
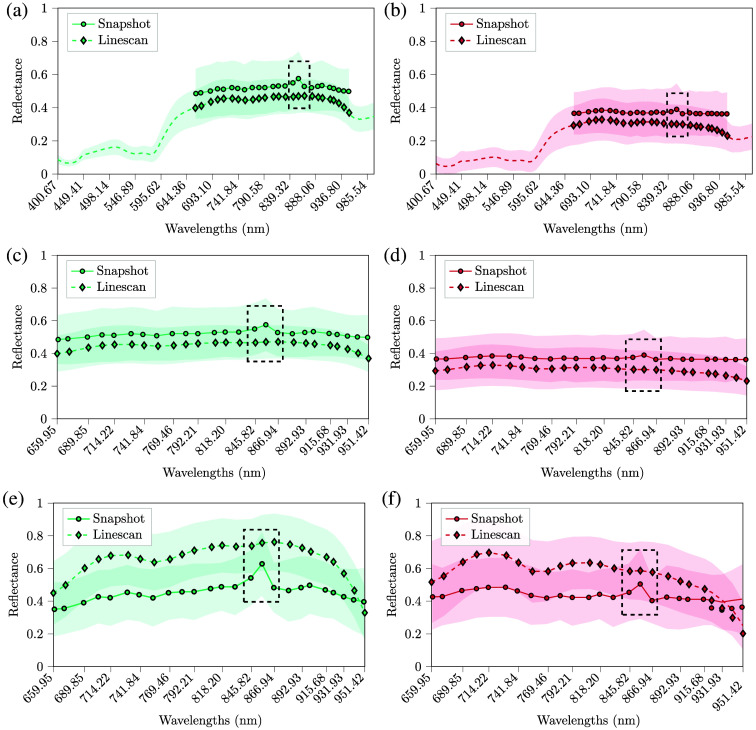
Mean reflectance spectral signatures with a standard deviation of all pixels included inside the 5×5 ROIs from both HS cameras. The shorter spectral signatures from 661.61 to 951.42 nm with continuous lines correspond to the snapshot camera in panels (a) and (b), whereas the longer spectral signature with dashed lines corresponds to the linescan camera. On one hand, plots in panels (c) and (d) are the same as panels (a) and (b), but with emphasis on the area common to both. On the other hand, panels (e) and (f) are the normalized spectra from panels (c) and (d). The dashed rectangles indicate the spectral bands of the HS snapshot camera influenced by the infrared of the depth camera. (a) Calibrated and denoised healthy tissue. (b) Calibrated and denoised pathological tissue. (c) Calibrated and denoised healthy tissue after band removal. (d) Calibrated and denoised pathological tissue after band removal. (e) Calibrated, denoised, and normalized healthy tissue after band removal. (f) Calibrated, denoised, and normalized pathological tissue after band removal.

To get an overview of the measurements, we illustrate in Fig. 6, the averaged results of all patients for both tissues presented in Fig. 6. The circular marks indicate the closest wavelengths between cameras specified in Table S1 in the Supplementary Material. By zooming into the common spectral range of the cameras between 659.95 and 951.42 nm from the previous plots, we present subfigures (c) and (d) to address in further detail the difference between measurements. Note the influence of the infrared peak emitted by the LiDAR at λ≈850  nm, illustrated in the dashed rectangles. Such a peak is only present in the snapshot measurements since the LiDAR could only be turned off when capturing with the linescan. By normalizing the pixels from subfigures (c) and (d), we obtained the mean spectral signatures with standard deviation for both tissues in subfigures (e) and (f).

**Fig. 7:**
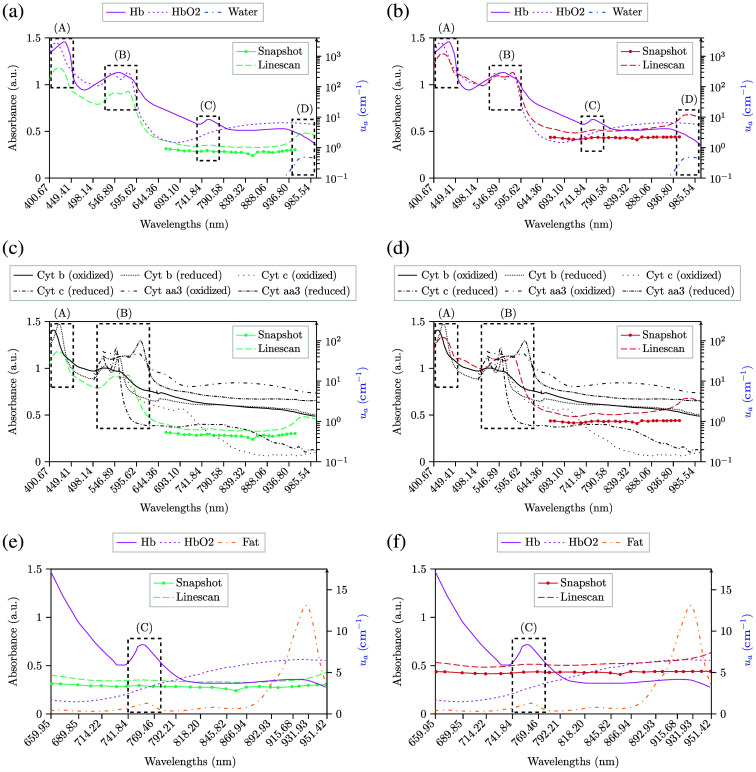
Mean absorbance spectral signatures of the 25 pixels included inside the rubber ring for both HS cameras. Data is calibrated and denoised. The shorter spectral signatures from 659.95 to 950.64 nm with continuous lines correspond to the snapshot camera, whereas the longer spectral signature with dashed lines corresponds to the linescan camera. The spectra of Hb and ${\mathrm{HbO}}_{2}$^33^ are shown in panels (a) and (b) with continuous and dashed magenta lines, respectively, whereas cytochromes b, c, and aa3^32^ are shown in panels (c) and (d) with different shades of oranges. Furthermore, continuous orange lines correspond to the absorption coefficient spectra of mammalian fat in panels (e) and (f).^34^ Hb, ${\mathrm{HbO}}_{2}$, Cyt. b, Cyt. c, Cyt. aa3, and fat are in ${\mathrm{cm}}^{-1}$, whose scale is in the right y-axis, whereas absorbance data measured with both cameras have its scale in the left y-axis. The A, B, and C dashed rectangles are used to indicate different absorption peaks of Hb, ${\mathrm{HbO}}_{2}$, Cyt. b, Cyt. c, Cyt. aa3, or fat, whereas the D rectangle points out a water absorption peak at λ=976  nm. (a) Healthy tissue. (b) Pathological tissue. (c) Healthy tissue. (d) Pathological tissue. (e) Healthy tissue after band removal. (f) Pathological tissue after band removal.

**Fig. 8:**
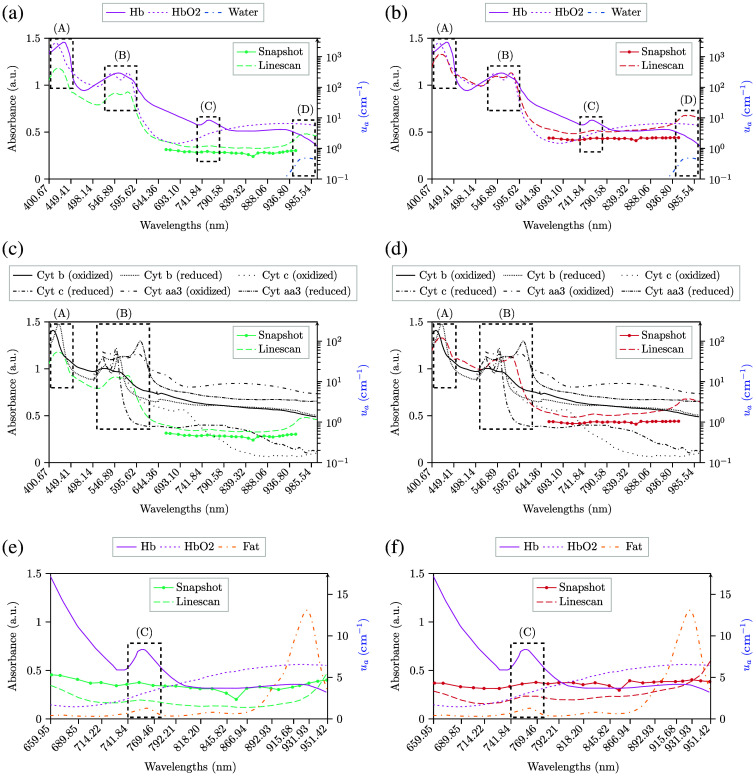
Mean absorbance spectral signatures of the 25 pixels included inside the rubber ring for both HS cameras. Data is calibrated, denoised, and normalized. The shorter spectral signatures from 659.95 to 950.64 nm with continuous lines correspond to the snapshot camera, whereas the longer spectral signature with dashed lines corresponds to the linescan camera. The spectra of Hb and ${\mathrm{HbO}}_{2}$^33^ are shown in panels (a) and (b) with continuous and dashed magenta lines, respectively, whereas cytochromes b, c, and aa3^32^ are shown in panels (c) and (d) with different shades of oranges. Furthermore, continuous orange lines correspond to the absorption coefficient spectra of mammalian fat.^34^ Hb, ${\mathrm{HbO}}_{2}$, Cyt. b, Cyt. c, Cyt. aa3, and fat are in ${\mathrm{cm}}^{-1}$, whose scale is in the right y-axis, whereas absorbance data measured with both cameras have its scale in the left y-axis. The A, B, and C dashed rectangles are used to indicate different absorption peaks of Hb, ${\mathrm{HbO}}_{2}$, Cyt. b, Cyt. c, Cyt. aa3, or fat, whereas the D rectangle points out a water absorption peak at λ=976  nm. (a) Healthy tissue. (b) Pathological tissue. (c) Healthy tissue. (d) Pathological tissue. (e) Healthy tissue after band removal. (f) Pathological tissue after band removal.

The analysis comparing the absorbance measurements using both HS cameras with the absorption coefficient spectra of deoxy-hemoglobin (Hb), oxy-hemoglobin (${\mathrm{HbO}}_{2}$),^33^ Cyt aa3, Cyt b, Cyt c,^32^ and mammalian fat^34^ is illustrated in Figs. 7 and 8. In addition, a detailed analysis of the results obtained with respect to the absorbance measurements is provided in Sec. S4 in the Supplementary Material, including Fig. S2 with all relevant absorption peaks for the chromophores under analysis.

### 3.3 Reflectance Spectral Similarities to Compare Both Cameras

**Fig. 9:**
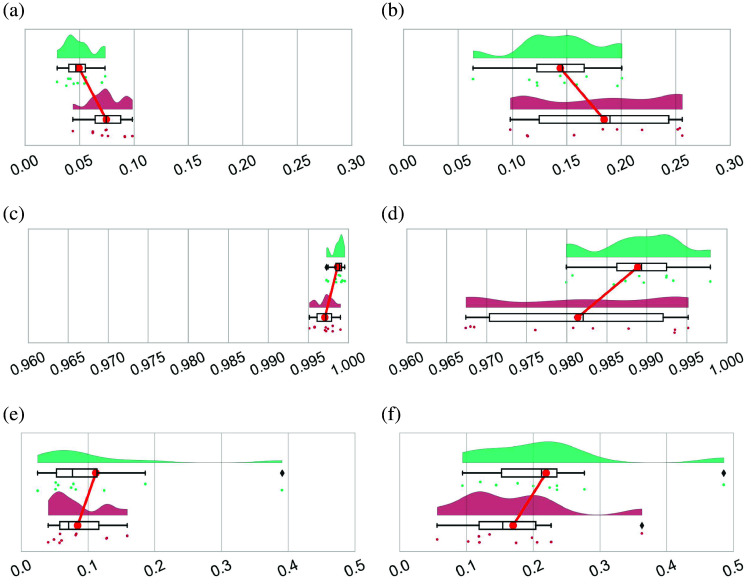
Raincloud plots with SAM, GFC, and RMSE similarity metrics. The left column plots [(a), (c), and (e)] are computed when pixels in the ROIs have been calibrated and denoised, whereas plots in the right column [(b), (d), and (f)] have additionally been normalized (after removing non-matched wavelengths of the HS linescan camera) using the min-max normalization from Eq. (3). Green distributions and dots indicate healthy tissue, whereas in red, they indicate pathological tissue. There is one dot in every distribution for every patient described in Sec. 2.4. Big red dots joined by a red line indicate the mean value of the distribution. (a) SAM metric for calibrated and denoised data. (b) SAM metric for calibrated, denoised, and normalized data. (c) GFC metric for calibrated and denoised data. (d) GFC metric for calibrated, denoised, and normalized data. (e) RMSE metric for calibrated and denoised data. (f) RMSE metric for calibrated, denoised, and normalized data.

The spectral similarities between the reflectances measured with both HS cameras are presented in Fig. 9. This figure shows raincloud plots^35^ for the healthy and pathological tissues with green and red colors, respectively. Raincloud plots are a useful graphical representation that addresses the challenge of data obfuscation in the presentation of error bars or box plots. These visualizations combine various data elements to display raw data points, probability density through half violin plots, and key summary statistics such as median, first and third quartiles, outliers with black diamonds, and relevant confidence intervals via boxplots. This combination produces a visually appealing and adaptable representation with minimal repetition. In addition, these plots have a red dot inside each box plot to illustrate the mean value of each distribution.

The pair of red dots shown in every plot of Fig. 9 are also connected to each other with a red line to visually see which distribution has a higher mean value. Furthermore, in these charts, each dot located below the box plots represents a single value of the corresponding similarity metric, which refers to the comparison of the mean spectral signatures for a particular patient and tissue type. For example, the leftmost green dot in Fig. 9(a) represents the SAM value of 0.029 obtained when comparing the healthy tissue of both cameras for the patient with ID 193.

For comparison purposes, each row in Fig. 9 shows a pair of raincloud plots for every metric described in Sec. 2.5. Plots located to the left of the figure are the similarity results computed when data were calibrated and denoised [Figs. 9(a), 9(c), 9(e), and 9(g)], whereas those to the right are obtained when data were additionally normalized [Figs. 9(b), 9(d), 9(f), and 9(h)]. The obtained metrics for the comparison of each patient, including both tissues under analysis, are presented in Table S2 in the Supplementary Material for further analysis. To evaluate the dispersion of the distributions, we will look at the interquartile range (IQR) which is computed as IQR=Q3−Q1, being Q1 and Q3 the first and third quartiles of the distribution, respectively. The IQR is a measure of the spread of data that is robust against extreme values. It provides valuable information about the variability of the central portion of a distribution and is helpful for identifying potential outliers. Besides, a comprehensive examination of the outcomes comparing the reflectance measurements from both cameras is presented in Sec. S5 in the Supplementary Material.

### 3.4 Identification of Chromophores in Absorbance Measurements

After studying the similarity of the reflectance measurements between the HS cameras in Sec. 3.2, we now attempt to identify the presence of any of the chromophores mentioned in Sec. 2.6 within the measured absorbances. Table S3 in the Supplementary Material presents SAM values resulting from the comparison between mean absorbance spectral signatures acquired using both HS cameras and the absorption coefficient spectra of the chromophores discussed in Sec. 3.2. The wavelength of the peaks of interest, the respective analyzed spectral ranges, and the number of bands considered are detailed. Notably, the comparison is made with data obtained from the linescan cameras in the VNIR, using all 369 wavelengths measured by the camera, and NIR regions for the snapshot and linescan cameras as well. For this latter case, we use the 25 overlapping bands between the two cameras, indicated in Table S1 in the Supplementary Material. To obtain the SAM values, we extracted the peak wavelength of interest and the fifteen wavelengths on each side of it for the chromophore under analysis. This ensures that the extracted bands correspond to the wavelength of interest and its nearby range. We have chosen to search for a maximum of 15 wavelengths on each side of the peak, as this allows us to extract spectral signatures with sufficient information. We then find the closest corresponding wavelengths between the selected chromophore bands and those measurable by each camera. It is important to note that it will not always be possible to use all 31 bands from the chromophore to identify a peak. This is because the spectral resolution of the cameras is lower than that used to measure the chromophores; hence, there will generally be fewer camera bands than those measured for each chromophore. For example, if the chromophore peak has a very narrow bandwidth, such as that from Cyt. b in its reduced state at λ=555  nm, there will not be many camera bands capable of measuring it. This also applies to searching for the closest snapshot camera wavelengths in the chromophores. In addition, we have included the mean spectral signatures of A measured by the cameras together with the $\mu_{a}$ spectral signatures of the chromophore peaks analyzed in Figs. S3–S18 in the Supplementary Material, allowing the reader to visually check the measurements.

## 4 Discussion

This work compares an HS snapshot camera with a linescan camera in the 659.95 to 951.42 nm range. Although similar studies have employed spectrometer measurements as a reference to address the comparison between HS cameras,^23^ using such an instrument was not feasible in this work because it would have required a sterilization process and placing the spectrometer close to an *in vivo* tissue during multiple surgical procedures. However, the effectiveness of the cameras was evaluated by conducting a comparison with the data obtained from a reference spectrometer over a standardized polymer reflectance. Pearson coefficients indicate a high correlation between the three systems and the given spectral response of the polymer provided by the manufacturer, with a value of around 96% for the linescan camera. Therefore, the linescan camera was considered to be the reference because it captures 15 times more spectral data and nearly four times more spatial data than the snapshot camera. Moreover, a similar linescan camera has already been used as an intraoperative tool for *in vivo* brain tumor classification with great results in the 400 to 1000 nm range.^15^ Visual analysis was made by comparing the mean spectral signature reflectance of both cameras in two scenarios, with and without data normalization. Furthermore, objective comparisons were made for both cases by computing four similarity metrics, SAM, GFC, and RMSE. Results have been illustrated in distributions to verify how they spread across the ten patients used for this study. We computed the IQR for each distribution to study the similarity of both cameras in the two previously described scenarios. In addition, we attempted to identify several chromophores in the absorbance measured with both cameras. The absorbances were obtained, for each patient, using the gathered reflectance as specified in Sec. 3.2.

We have observed that the snapshot camera measurements are noisier compared with those from the linescan camera. Regardless of the laser emission at λ≈850  nm from the LiDAR of the acquisition system, which must be turned on to visualize a live video during surgery, the spectral signatures appear less smooth than those obtained with the linescan camera. This is evident from the mean reflectance spectral signature of the snapshot, which shows several peaks and valleys along the spectral range from 659.95 to 950.64 nm, which could be due to the noise introduced by the sensor. In fact, the Fabry-Pérot sensor of the snapshot with 25 filters has been characterized in detail by Hahn et al.,^22^ concluding that the correction matrix provided by the manufacturer is insufficient to reconstruct the spectrum without introducing large measurement errors. Furthermore, the irregularities found in the sensor are present across the whole sensor, hence, in the entire spectral range of the camera. Although Hahn et al. propose to create an individual matrix after characterizing the camera, a dedicated optical system is required which was not available for this study. To mitigate this issue, previous works by Muhle et al. using the same snapshot camera model averaged 10 captures for organ transplantation purposes.^23^ However, we could not adopt the same method for imaging *in vivo* tissue because multiple captures would yield varying spatial measurements due to the brain movement caused by the heartbeat. Despite the noise introduced in the snapshot camera measurements, the trends of the spectral signatures are similar to those obtained with the linescan camera. In general, both cameras seem to agree that the pathological tissue provides less reflectance than healthy tissue when data is unnormalized. Such behavior can be seen in the two first columns of Fig. 5.

The errors in the snapshot camera measurements are more pronounced in the spectral signatures in Fig. 5 once the data is normalized. This was expected since a min-max normalization was applied to each spectral signature individually, causing errors in measurements to increase since data is scaled from the 0 to 1 range. This behavior is illustrated within the spectral signatures in the two right columns of Fig. 5, where the continuous lines have a noisier trend than the dashed spectral signatures coming from the linescan camera. In consequence, normalized data from both cameras appear to be less similar to each other than when reflectance data is unnormalized. This fact is confirmed after analyzing the distributions with the SAM, GFC, and RMSE metrics, which are used to address the comparison of the cameras. Generally, distributions using normalized data have higher IQR values since they are more spread than those distributions with unnormalized data. For example, IQR values from unnormalized data for the healthy tissue are 0.023, 0.001, and 0.061 for the SAM, GFC, and RMSE metrics, respectively, whereas the results from normalized data are 0.119, 0.006, and 0.083 for the same metrics.

Expected differences in reflectance intensity, as seen in Fig. 5, could also arise due to variations in lighting angle and working distance. These variations are traditionally corrected using a white reference image [$I_{\text{white}}$ from Eq. (1)], obtained from a flat calibration board.^15^^,^^18^ However, the 3D structure of the brain and the inherent organ texture variations can cause discrepancies in spectral characteristics due to deviations in illumination and working distance across the surface.^36^

Although unnormalized data make spectral measurements very similar for both cameras, as presented in Figs. 7(e) and 7(f), the spectra is very steady throughout the spectral range under analysis. Hence, the presence of absorption peaks, such as the one that Hb has at λ=756  nm, might be hardly noticeable with unnormalized data. Normalized data seem to illustrate better the presence of Hb or blood with local minimums at λ≈756  nm, as presented in Figs. 8(e) and 8(f). This behavior is found when analyzing the SAM values in Table S3 in the Supplementary Material to identify such absorption peaks in the absorbance of the cameras, where normalized data has 0.03 less SAM than that obtained with unnormalized data, regardless of the tissue. However, the SAM values obtained with the snapshot cameras are higher with normalized data than with unnormalized data. This might be due to the noise introduced by the sensor, as already explained previously. Moreover, observations show how the contribution of Hb is slightly higher in pathological tissue than in healthy tissue at λ=756  nm, which may be related to increased perfusion of tumor tissue, especially in high-grade tumors, or may even be related to lack of oxygen to brain tissue or tumor hypoxia due to abnormalities in tumor vessel structure.^37^ Such behavior was found in other studies using HSI for *in vivo* human brain^29^ and might also indicate hypoxia from glioma cells.^38^ The analysis conducted in Sec. 3.4 aimed to identify any chromophore absorption peaks in the absorbance measurements of the cameras. The results indicate overoptimistic SAM values for most peaks, which do not seem to correlate with the spectra in Figs. S3–S18 in the Supplementary Material. However, the absorbance measurements of the linescan camera might indicate the presence of four peaks, as shown by the SAM values in Table S3 in the Supplementary Material and their corresponding spectra in Figs. S4, S13, S14, and S18 in the Supplementary Material. These peaks correspond to an absorption peak of oxidized Cyt. b at λ=422  nm, two peaks of ${\mathrm{HbO}}_{2}$ at λ=542  nm and λ=576  nm, and the water absorption peak at λ=976  nm. Regardless of the tissue under analysis and the data used, the values we obtained are SAM≈0.235 for the peak at λ=422  nm, SAM≈0.210 for the peak at λ=542  nm, SAM≈0.110 for the peak at λ=576  nm, and SAM≈0.235 for the peak at λ=976  nm. This statement makes sense because the first three peaks have the highest absorption coefficient values, which means they could potentially be measured. Although these peaks are the most absorbent and might help during tumor detection, further research is needed to evaluate if the snapshot camera employed can detect pathological tissue in the 659.95 to 950.64 nm range.

## 5 Conclusions

In this study, we have compared and analyzed two different HS cameras because of their potential to be used for intraoperative brain tissue identification. Specifically, we have used images from ten *in vivo* human patients with different pathologies. Measurements show how the snapshot camera with less spectral and spatial resolution can capture a similar spectral behavior than that obtained with the linescan camera. Although the linescan camera has almost three times more spectral resolution than the snapshot, it required 1 min and 40 s to scan a single *in vivo* human brain image. Hence, it is not suitable for real-time solutions compared with the snapshot camera that requires 100 ms to acquire the data. Furthermore, such camera has already been used in real-time solutions for *in vivo* human brain tumor classification,^19^ acquiring and processing the HS data at 14 frames per second. Moreover, objective comparisons were made in the shared spectral range of both cameras between 659.95 and 951.42 nm using four similarity metrics: SAM, GFC, and RMSE. Results with unnormalized data show high similarity between the reflectances captured with the cameras in the aforementioned spectral range for either healthy or pathological tissues. However, due to the noise introduced by the snapshot mosaic sensor,^22^ the similarity between cameras is reduced once data is normalized. For example, the SAM metric shows that there is reduced dispersion and high similarity between cameras for pathological samples, with an IQR value of 9.68% for normalized data, compared with an IQR value of 2.38% for unnormalized data. This behavior is consistent also for both GFC and RMSE, irrespective of the type of tissue under inspection. Differences in similarity between cameras may be attributed to errors that arise from the independent normalization applied to each spectral signature to minimum and maximum values between 0 and 1. Even though noiseless measurements from the snapshot camera could be obtained by averaging multiple images,^22^^,^^23^ such procedure is not feasible during *in vivo* brain surgeries due to the heart beating, which pumps blood to the brain causing it to move. Furthermore, we studied the ability of both cameras to identify several tissue chromophores in their measurements. In particular, we attempted to identify Hb, ${\mathrm{HbO}}_{2}$, fat, water, and several cytochromes by converting the measured reflectance of the cameras to absorbance, as specified in Sec. 3.2. Such task is done by trying to identify relevant peaks of the previous chromophores. For that, we apply the SAM metric as it is the only one that considers the shape of the spectra. Furthermore, the identification of chromophores was also conducted through a subjective inspection of the absorbance spectra measured with the cameras in comparison to the absorption coefficients of the chromophores. Out of the 21 peaks analyzed, only five could potentially be identified by the snapshot camera as most of them are present in the visible spectrum, specifically from the 400 to 625 nm spectra. However, the snapshot camera encountered difficulties in identifying any of those five peaks, which are from oxidized Cyt. c at λ=695  nm, Hb at λ=756  nm, and three fat peaks at λ=756  nm, λ=830  nm, and λ=930  nm. Such difficulties could be due to their low absorption coefficient values compared with those in the visible spectrum and the low spectral resolution of the camera. Nonetheless, we observed that the linescan camera detected four absorption peaks, which corresponded to three different chromophores present in its absorbance measurements. These peaks correspond to the oxidized Cyt. b peak at λ=422  nm, to two peaks of ${\mathrm{HbO}}_{2}$ at λ=542  nm and λ=576  nm, and to a water peak at λ=976  nm. Regardless of the tissue and data used, the obtained SAM values between the absorbance of the camera and the absorption coefficient of the chromophores were approximately 0.235, 0.210, 0.110, and 0.100, respectively. These values suggest high similarities between the spectra and the possible presence of the mentioned chromophores in the absorbance measurements.

All things considered, the snapshot camera can provide reasonable measurements to describe brain tissue behavior when compared with the typical linescan cameras used for brain tumor detection.^15^^,^^29^^,^^39^^,^^40^ Likewise, the snapshot camera offers great opportunities to provide real-time solutions as employed in other studies.^19^ However, combining multiple snapshot cameras to increase the spectral range can lead to a better reconstruction of the spectral behavior of biological tissues as shown in other works.^23^ Therefore, this study shows the potential use of snapshot cameras for *in vivo* brain tissue identification. Moreover, similar spectral measurements from both cameras were obtained, suggesting the combination of data from both cameras to train classification models and enhance *in vivo* brain tumor classification.

## Supplementary Material



## Data Availability

All the *in vivo* hyperspectral human brain data used in this study are from the Slim Brain database, which is available at https://slimbrain.citsem.upm.es/. Note that access must be granted, under reasonable request, before downloading the data.
